# An all-sputtered photovoltaic ultraviolet photodetector based on co-doped CuCrO_2_ and Al-doped ZnO heterojunction

**DOI:** 10.1038/s41598-021-98273-5

**Published:** 2021-09-21

**Authors:** Morteza Ahmadi, Masoud Abrari, Majid Ghanaatshoar

**Affiliations:** 1 Laser and Plasma Research Institute, Shahid Beheshti University, Tehran, Iran; 2 Solar Cell Research Group, Shahid Beheshti University, Tehran, Iran

**Keywords:** Sensors, Solar cells, Optical materials and structures

## Abstract

We propose and fabricate a heterojunction between Al-doped ZnO and (Mg, N)-doped CuCrO_2_ thin films using the sputtering deposition method. These materials possess wide bandgap that makes them transparent in the visible light but excellent UV-absorbers. On the other hand, the high conductivity of these materials, respectively as n-type and p-type transparent conducting oxides, facilitates the charge transport. We show that the p–n junction fabricated from these materials has the potential to act as a high-performance UV photovoltaic photodetector. The proposed structure, demonstrates fast responses in order of sub seconds, photosensitivity of ~ 41,000, responsivity of 1.645 mA/W, and a detectivity of 3.52 × 10^12^ Jones that are significantly improved in comparison with the Al-doped ZnO photoconductor. This excellent improvement is attributed to the capability of the photovoltaic configuration that creates a built-in voltage and facilitates the charge separation and collection rather than recombination in the photoconductor configuration.

## Introduction

Photodetectors are devices that sense photons with specific wavelengths and convert them to electrical signals via photon/matter interaction^1^. They are an essential branch of optoelectronics with numerous applications, including photography, spectroscopy, characterization, optical communications, range finders, etc.^2–5^. Each of these applications becomes possible by working in distinct wavelength ranges. For instance, photography is performed by the detection of photons in the visible spectrum, while the infrared spectrum is used for spectroscopy and long-distance optical comminutions due to its low absorption in air. However, photodetection in the ultraviolet (UV) region has been less-exploited than the regions mentioned above due to its lesser-known applications. Recently, scientists have introduced novel applications for UV photodetectors which include non-line-of-sight optical communication^6,7^, non-destructive testing^8,9^, observation of hot stars in astronomy^10,11^, air-pollution monitoring^12^, etc., that have attracted huge attention to UV photodetectors.

Zinc oxide (ZnO) with a wide bandgap of > 3.1 eV, a high electron mobility of ~ 200 cm^2^V^−1^ s^−1^, and an exciton binding energy of ~ 60 meV has proven to be an outstanding material for UV detection^13,14^. It has been studied in both forms of photoconductive and photovoltaic structures for UV sensing. Yet, its relatively poor electrical conductivity has made the researchers either implement it in lower-dimension nanostructures such as nanowires, nanorods, or core-shells to conduct the charge carriers in the desired direction^15,16^ or add dopants like aluminum or gallium to its lattice to increase its conductivity^17,18^.

Although the modified ZnO-based UV photodetectors have shown outstanding performance in the photoconductive mode, their photovoltaic structures are better anticipated due to their higher efficiencies and lack of requirement for external power source ^19,20^. In photovoltaic designs, a p–n junction with a suitable band-structure is formed, which generates an internal (built-in) voltage that can separate the electron–hole pairs produced by photon absorption. However, an external negative bias is usually applied to the photovoltaic photodetectors so that their space-charge region is widened that reduces the reverse saturation current, and further increases the sensitivity and responsivity of the device^21,22^. In 2005, Moon et al. fabricated a ZnO-based photovoltaic UV photodetector with ZnO and arsenic-doped ZnO respectively as n-type and p-type materials, which yielded a photocurrent of ~ 2 mA at a wavelength of 325 nm and biasing voltage of − 3 V^23^. Five years later, their work was improved by Leung et al. by using (Al and N) co-doped ZnO as the p-type layer. In more recent studies, Ning et al. and Wang et al. could further enhance the photodetection performance of ZnO homojunction by employing nanostructures and respective use of silver and antimony dopants^24,25^. Yet, heterostructure photodetectors have demonstrated superior performances. For example, ZnO has been used as the n-type material along with GaN^26^, CuSCN^27,28^, NiO^29^, perovskites^30^, Ga_2_O_3_^31^, and Cu_2_O^32,33^ p-type nanostructures and responsivities of up to 60 mA/W have been achieved. In an exciting work, Coussuet et al. showed that a ZnO p–n junction with CuCrO_2_ p-type delafossite in core–shell structure has a high potential for UV photodetection with a fast response in order of microseconds and responsivity of 5.87 mA/W at 395 nm wavelength, which demonstrated the excellent properties of delafossite materials for UV detection^15^. All the aforementioned UV photodetectors were based on nanostructures that are more challenging for fabrication and practical applications in comparison with simple p-n thin films.

Delafossite is a family of materials with a wide bandgap that demonstrates p-type conductivity. These materials were first introduced as p-type transparent conducting oxides (TCO) in 1997 by Kawazoe et al., which revealed the p-type conductivity of the CuAlO_2_ thin films^34^. Among delafossite materials, CuCrO_2_ has shown the best TCO properties, which are due to its large bandgap of ~ 3 eV and its higher delocalization of holes produced by Cu vacancy that creates shallow energy transition levels for them by mixing of Cr 3d and O 2p states in the valence band^35,36^. CuCrO_2_ properties are further improved by addition of dopants such as Mg, Zn, Ca, Al, etc.^37^ and yielded the best results with Mg doping by Nagarajan et al. in 2001^38^. Moreover, CuCrO_2_ has been prepared by both chemical and physical methods, including metal–organic chemical vapor deposition^39^, spray pyrolysis^40^, sol–gel^41^, pulsed laser deposition^42^, and different kinds of the sputtering method^37,43–45^. Among these methods, radio-frequency (RF) magnetron sputtering has yielded the best results so far^38,46^.

In our previous studies, we have prepared both high-quality ZnO and CuCrO_2_ thin films^46,47^. Regarding ZnO thin film, we could enhance its electrical conductivity through the addition of Al dopant and the creation of oxygen vacancies in its lattice through the vacuum heat-treatment of the target prior to deposition^47^. In the case of CuCrO_2_, we improved its properties by simultaneous doping with Mg and N through reactive sputtering, which could achieve better conductivity compared to previous works^46^. We believe that the fabrication of a heterojunction device between these two improved thin films can perform very suitable as a UV photodetector without the need for more complex nanostructures. These materials have proven to possess high UV photonic absorption which is required for electron–hole generation. On the other hand, their improved conductivity and suitable band-alignment will provide electronic pathways and enough built-in voltage to facilitate the charge transport in the device, which results in a photovoltaic effect. Therefore, we can achieve a high-performance UV photodetector with high visible-light transparency, good responsivity, and appropriate detectivity in the thin-film form that can be deposited for practical applications. Thus, in the current study, to achieve a high-performance UV photodetector, we fabricate a p–n junction of Al-doped ZnO (AZO) as the n-type and (Mg, N) co-doped CuCrO_2_ as the p-type materials and analyze its UV-detection properties. Both layers are deposited through the RF magnetron sputtering method, and Au/Ag layers are used to achieve ohmic contacts. We also prepare an AZO photoconductor for comparison and to demonstrate the role of p-n device fabrication in the enhancement of the photodetector properties.

## Results and discussion

**Figure 1:**
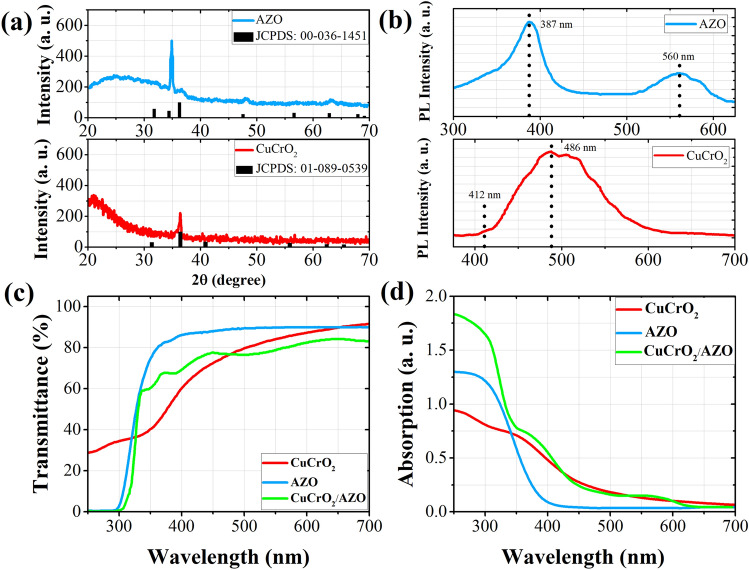
(**a**) Grazing XRD patterns of the prepared co-doped CuCrO_2_ thin film after the post-deposition annealing and AZO thin film without post-deposition annealing, deposited on quartz substrate along with the corresponding JCPD references. (**b**) PL spectra of the AZO and co-doped CuCrO_2_ thin films. (**c**) Transmittance and (**d**) absorption spectra of the prepared layers and the fabricated device acquired with UV–vis analysis.

We employed X-ray diffraction (XRD) analysis to find out about the crystallinity of our prepared thin films. The results are shown in Fig. 1a. As we can see for the co-doped CuCrO_2_ sample, there exists a single sharp peak at around 2θ = 36°, which corresponds to the rhombohedral structure oriented alongside the (012) plane of the delafossite phase (JCPDS:01–089–0539). The noisy background with a vast peak is due to the amorphous nature of the employed quartz substrates. Furthermore, for the AZO thin films deposited on the quartz substrate, we observe a very distinct peak at around 2θ = 35° that shows the preferred orientation along the (002) plane of wurtzite structure (JCPDS:00-036-1451). These sharp peaks in the XRD patterns of the prepared thin films, which demonstrate a preferential growth, can be considered as the result of the surface energy minimization when the interfacial energy between the substrate and nuclei can be ignored. In other words, after the formation of the nuclei with random orientation, surface diffusion promotes the preferred orientation of the nuclei to the crystallographic direction with the lowest surface energy^48^. In our case, (012) and (002) planes have the lowest surface energies and smaller activation energies for nucleation and faster growth velocity in co-doped CuCrO_2_ and AZO thin films, respectively. The average crystallite sizes of our prepared thin films were estimated through the Debye–Scherrer formula to be ~ 18 nm and ~ 20 nm for CuCrO_2_ and AZO layers, respectively.

Photoluminescence (PL) spectroscopy is among the most capable tools for optical analysis of the delafossite materials. Figure 1b shows the PL spectra of our prepared AZO and co-doped CuCrO_2_ samples. In the AZO spectrum, there is an almost sharp peak at around 387 nm that reveals the near band edge (NBE) emissions in UV region. Typically, NBE emissions are attributed to band-to-band transitions or excitonic recombination^49^. Therefore, the energy bandgap of our prepared AZO thin film will be approximately 3.2 eV. This value is close to our previous work results, which was between 3.15 to 3.17 eV^47^. Carey et al. also measured a bandgap of 3.2 ± 0.3 eV for AZO layer deposited by magnetron sputtering on β-Ga_2_O_3_ substrate^50^. There is also a vast peak witnessed at around 560 nm wavelength which we can correspond it to the oxygen deficiencies in the AZO crystalline structure^51^. The visible peak to the UV peak ratio is an illustrator of the oxygen deficiencies in AZO material. In the case of a stochiometric ZnO, we will only observe a single PL peak in the UV region. The appearance of two peaks in the prepared AZO thin films, demonstrates the existence of oxygen deficiencies which is a result of high vacuum treatment of AZO sputtering targets^47^. Furthermore, in the PL spectrum of the co-doped CuCrO_2_ thin film there exists a shallow peak around 412 nm that demonstrates the band-to-band transitions in this sample. Based on this peak, which indicates the threshold of optical bandgap, the bandgap of CuCrO_2_ is estimated to be 3.01 eV, which is very close to the values measured by Kaya et al. (3.00 to 3.07 eV for various Mg-doped samples)^52^. Tripathi and Karppinen also reported a bandgap of 3.0 eV with 2.5% Mg doping for CuCrO_2_^53^. The wide and high peak between 450 to 550 nm wavelengths, can generally be corresponded to the defects in CuCrO_2_ lattice. CuCrO_2_ is an intrinsic p-type semiconductor which its p-type conductivity is owed to the Cu vacancies in its lattice and is further intensified by the Mg and N dopants^46^. Such behavior has been previously observed and confirmed in CuAlO_2_ delafossite material^54,55^. The other peak witnessed at 486 nm can be attributed to Cu deficiencies in CuCrO_2_ lattice which act as trap states inside the bandgap^56^.

The optical transmittance spectra of the AZO, co-doped CuCrO_2,_ and their multilayer are illustrated in Fig. 1c. In this analysis, both the p-type and n-type layers demonstrate a high transmittance of more than 90% in the visible region, which displays their high transparencies. These high transparencies can be acquired when the materials are in their crystalline forms that we observed in the XRD analysis. The multilayer device also exhibits high transparency, but its value is lower than the individual layers. The high transmittance of ~ 85% for the bilayer demonstrates its high transparency in the visible region and is promising for the fabrication of transparent photodetectors. We also witness some ripples in its spectrum which are the result of the bilayer interface and the higher thickness of the bilayer in comparison with each separate layer. The light absorption was also investigated and displayed in Fig. 1d, in which we witness high absorption in the UV region that emphasizes the suitability of these materials and the bilayer for UV detection. Moreover, we see two distinct peaks in the absorption spectrum of the AZO/CuCrO_2_ bilayer, which correspond to the slightly different absorption edges of AZO (~ 360 nm) and co-doped CuCrO_2_ (~ 380 nm). These results indicate that the employed materials and the fabricated p–n junction have an excellent selectivity in the UV region and can be very promising for application as transparent UV photodetectors.

**Figure 2:**
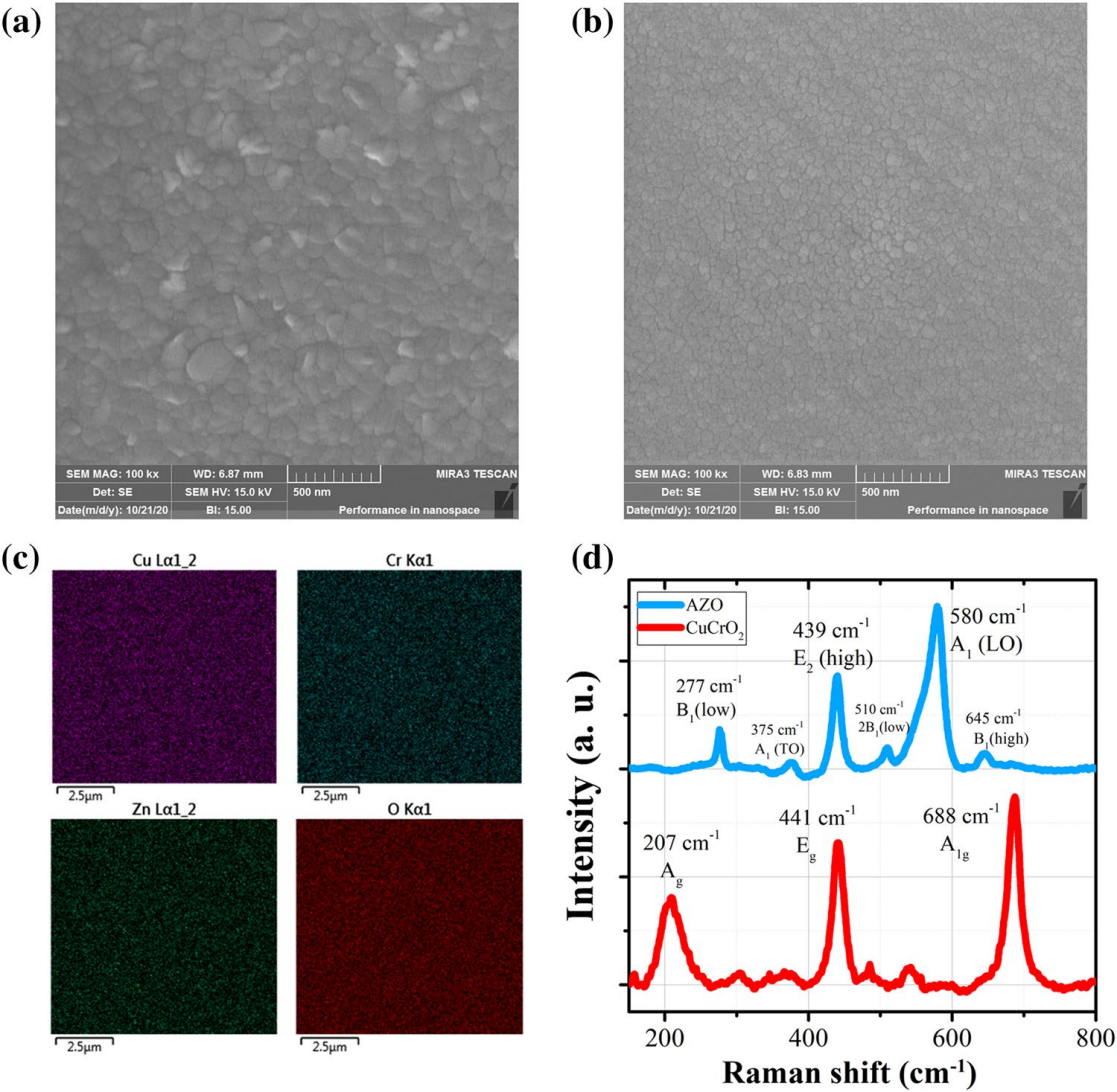
Top view FESEM images of (**a**) AZO and (**b**) co-doped CuCrO_2_ layers. (**c**) EDS mapping analysis of the primary elements in our prepared samples. (**d**) Raman scattering spectra of the deposited AZO and co-doped CuCrO_2_ layers.

We investigated the morphology and surface properties of our prepared materials using FESEM analysis. The micrographs of co-doped CuCrO_2_ layer deposited on quartz substrate and AZO layer deposited on top of the CuCrO_2_ layer are shown respectively in Fig. 2a,b. In these images, we observe a granular morphology with densely packed nanoparticles for both CuCrO_2_ and AZO thin films, with the latter having larger grain sizes. It is also shown that the prepared thin films are free of macroscopic cracks and deformities and possess excellent homogeneity, compactness, and uniformity that demonstrate the appropriate thin film deposition through the sputtering method. This homogeneity and suitable layer formation are crucial in the materials’ electronic and optical properties. We also analyzed the elemental composition of the prepared thin films using energy dispersive X-ray spectrometry (EDS). Supplementary Table S1 shows the atomic ratios of the prepared thin films. We can deduce from the table that both the AZO and co-doped CuCrO_2_ materials are in a nearly stochiometric balance. There are minor variations from the stochiometric states in these materials which are due to the oxygen vacancies in AZO and Cu vacancies in co-doped CuCrO_2_ materials. These vacancies were previously seen and confirmed in the PL analyses., The elemental mapping results for Zn, Cu, Cr, and O elements are displayed in Fig. 2c. We see from these results that all the elements have a homogenous distribution along the deposition area which again confirms the successful preparation of the desired materials. The EDS mapping results of the Al dopant in ZnO and Mg and N dopants in CuCrO_2_ lattices are delivered in the Supplementary Fig. S1. In this figure, the existence and incorporation of the desired dopants in the ZnO and CuCrO_2_ lattices are confirmed. The EDS mapping results of the dopants show lower densities in comparison with the mapping of Zn, O, Cu and Cr elements that is due to their lower concentrations in the samples.

We employed Raman analysis for a more in-depth analysis of the prepared layers’ structure. Figure 2d illustrates the Raman scattering spectrum for AZO and co-doped CuCrO_2_ materials. As we are aware, the delafossite structure (ABO_2_) belongs to the R-3 m space group and can be irreducibly addressed by^57^:

$$\Gamma ={\mathrm{A}}_{\text{1g}}\text{ + }{\text{E}}_{g}\text{ + 3}{\text{A}}_{\text{2u}}\text{ + 3}{\text{E}}_{u}.$$

There are a total of 12 vibrational modes, of which 3 are acoustic and 9 are optical modes. However, only ${\text{E}}_{\mathrm{g}}$ and ${\mathrm{A}}_{\text{1g}}$ are active Raman modes while ${\text{E}}_{\mathrm{u}}$ and ${\text{A}}_{\text{2u}}$ correspond to the active infrared modes. Nevertheless, $\mathrm{A}$ modes are the ones in which the atomic vibrations are in the c-axis (the Cu–O bond in CuCrO_2_ structure), and ${\text{E}}$ modes are the modes perpendicular to the c-axis (a-axis). In this condition, there is a dual degeneracy for ${\text{E}}$ modes in the a-axis due to the vibrations in two perpendicular directions. We can deduce from the Raman spectrum that the peaks located at 207, 441, and 668 cm^−1^ respectively demonstrate ${\mathrm{A}}_{\text{g}}$, ${\text{E}}_{\mathrm{g}}$_,_ and ${\mathrm{A}}_{\text{1g}}$ vibrational modes^58,59^. Considering these vibrational modes, we can confirm the formation of the delafossite phase with rhombohedral structure in our prepared thin films. The Raman spectrum of the AZO material show distinct peaks at around 277, 375, 439, 510, 580, and 645 cm^-1^ that are matched with ${\mathrm{B}}_{1}$ (low), ${\mathrm{A}}_{1}$ (TO), ${\mathrm{E}}_{2}$ (high), 2 ${\mathrm{B}}_{1}$ (low), ${\mathrm{A}}_{1}$ (LO), and ${\mathrm{B}}_{1}$ (high) vibrational modes, respectively, that further confirms the formation of AZO hexagonal phase^60–62^.

**Figure 3:**
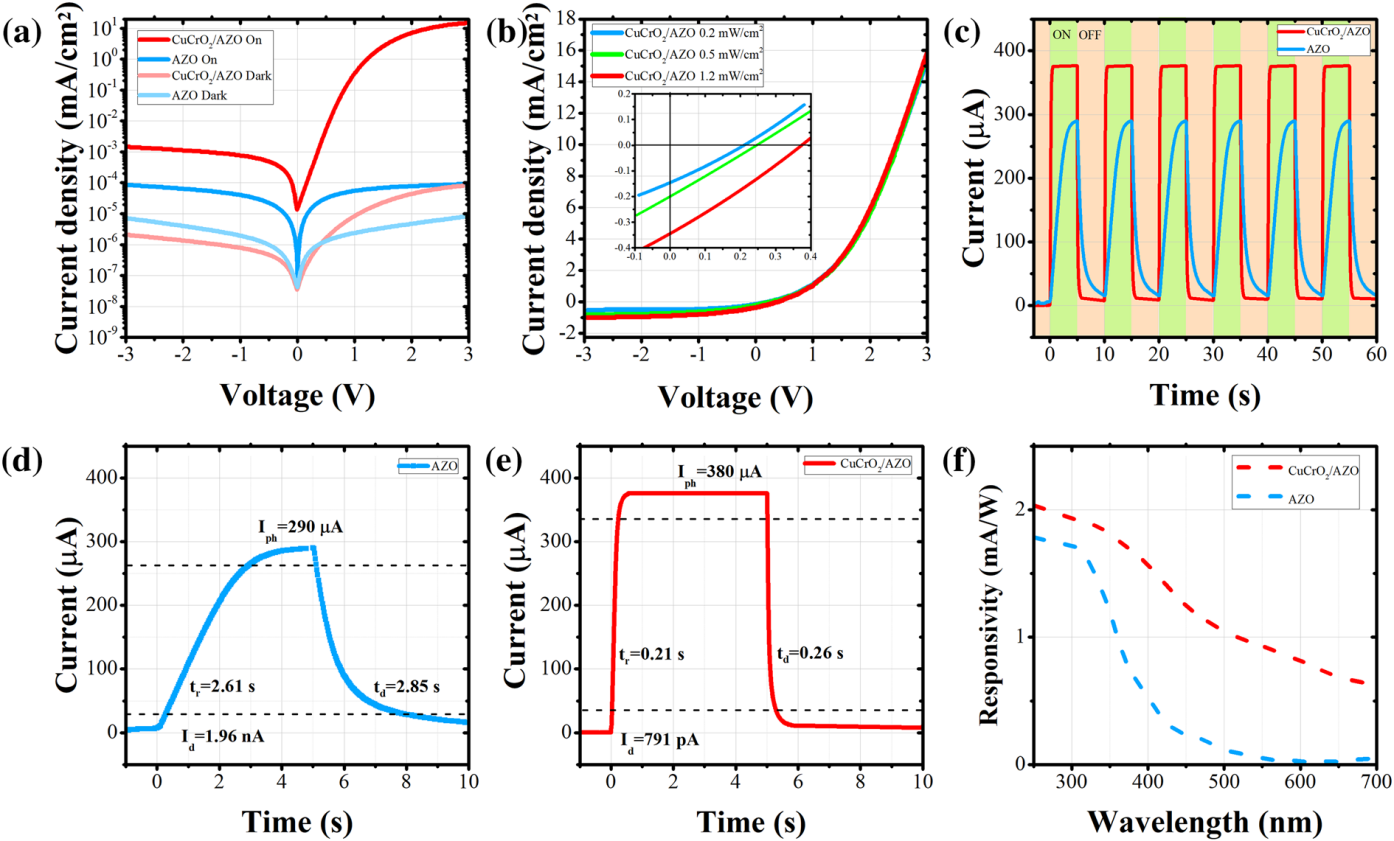
(**a**) Semilogarithmic plot of the J–V characteristics of the fabricated photovoltaic and photoconductive UV photodetectors in dark and illuminated conditions. (**b**) J–V characteristics of the AZO/co-doped CuCrO_2_ p–n junction under 0.2, 0.5 and 1.2 mW/cm^2^ illumination intensities and the zoomed curve around zero biasing (inset). (**c**) Current–time (I–t) measurement of the photovoltaic and photoconductive photodetectors under periodic illumination by a 385 nm wavelength and 0.2 mW/cm^2^ intensity LED. (**d**) Transient behavior of the AZO layer and (**e**) the fabricated p–n junction under UV illumination. The dashed lines represent the 10% and 90% of maximum values in which the rise and decay times are measured in between, respectively. (**f**) Spectral responsivity of the AZO photoconductive and AZO/co-doped CuCrO_2_ photovoltaic photodetectors.

In order to investigate the photodetection properties of the prepared devices, we employed various current–voltage and current–time measurements and illustrated their results in Fig. 3. The photodetection of a single layer co-doped CuCrO_2_ in photoconductive configuration was also investigated, but its results are illustrated in Supplementary Fig. S2 and Supplementary Fig. S3 due to its lower performance. Figure 3a shows the current density–voltage (J–V) curves of the AZO and co-doped CuCrO_2_/AZO bilayer devices on a semilogarithmic scale. In the photoconductor configuration (single AZO layers with contacts), we observe a symmetrical behavior in the J–V characteristic, which confirms the successful fabrication of ohmic contact between Au/Ag and AZO layers. Furthermore, after illumination by an LED source with a wavelength of 385 nm and an intensity of 0.2 mW/cm^2^, we witness a dramatic increase in the current density from 2.4 nA/cm^2^ to 56 nA/cm^2^ at 1 V bias. It shows an on/off ratio of ~ 24 and confirms the excellent quality of AZO material to be used as a photoconductive photodetector. By adding a co-doped CuCrO_2_ layer in the photovoltaic configuration, the symmetrical behavior of the J–V curve is changed, which is due to the rectification behavior of the p–n junction that approves the successful fabrication of heterojunction and their appropriate band alignment. Moreover, we observe a significant increase in the photogenerated current of the device by illuminating it by a UV source. This is attributed to the formation of a depletion layer with the built-in voltage that facilitates the charge separation and collection rather than its recombination. We also witness a dramatic growth in the device current density from 8.36 nA/cm^2^ to 346 µA/cm^2^ at 1 V biasing voltage after illumination that demonstrates the excellent performance of the device as a photovoltaic UV photodetector with ~ 41,000 on/off ratio. Figure 3b shows the typical plot of the J–V measurement carried out on the photovoltaic configuration under different illuminations of 0.2, 0.5, and 1.2 mW/cm^2^. In this figure, the diode-like behavior of the device is clear. Furthermore, we can evidently see that at reverse bias voltages (that is mostly used in photodetectors to expand the depletion region in order to acquire higher absorption and internal potential), the amount of photogenerated current is increasing by the increase in the incoming illumination power. At V = − 1 V, the amount of photogenerated currents (photoresponse) of the device for 0.2, 0.5 and 1.2 mW/cm^2^ UV illuminations are respectively 451, 612 and 770 µA/cm^2^. This result confirms that the photoresponse of the charge carriers in our device is proportional to the incoming photon flux with a power-law relation^63^:

$${I}_{ph}=A{P}^{\theta }$$

in which ${I}_{ph}$ refers to the photogenerated current, $P$ stands for the illumination intensity, $A$ is a constant corresponding to the wavelength, and $\theta$ has a value between 0.5 and 1.

In high-performance photodetectors, the reproducibility of the photoresponse is very important. We have examined this parameter by investigating the current–time (I–t) characteristics of our prepared devices that are shown in Fig. 3c for periodic illumination with a 385 nm and 0.2 mW/cm^2^ UV source. These devices exhibit an excellent reproducibility without noticeable variations. In the photoconductor configuration, the photoresponse slowly increases with a ramp during the on-time to ~ 290 µA and again starts decreasing gradually to its initial state after turning off the illumination, generating a triangular-like waveform. By adding a co-doped CuCrO_2_ layer to this device, the electron–hole pair separation speeds up as a result of the depletion region formation, and we witness a much sharper photoresponse in the photovoltaic configuration. In this condition, the photoresponse demonstrates a square-like waveform. These results once again confirm the effectiveness of adding a co-doped CuCrO_2_ layer to the AZO for UV photodetection.

The transient behavior of the devices was investigated with more details in Fig. 3d,e, in which the rise time (t_r_) and decay time (t_d_) of the devices are emphasized. We observe a t_r_ of 2.61 s and a t_d_ of 2.85 s for the photoconductor configuration while these values are reduced to t_r_ = 0.21 s and t_d_ = 0.26 s for the photovoltaic configuration, indicating a much faster response time for the fabricated p–n junction. The faster response times of the AZO/co-doped CuCrO_2_ heterojunction in comparison with the Au/AZO/Au structure can be justified by both semiconductor physics and electronic phenomenon^64^. The photoresponse of the device in metal/semiconductor/metal structure is due to the diffusion current between two electrodes while in the photovoltaic configuration, the photoresponse is determined by the drift velocity of the p–n junction which is faster than the diffusion velocity. In other words, in the photoconductive configuration, the response is limited by the change in the resistivity as a result of the photogenerated charge carriers while in the photovoltaic configuration, it is the RC time constant of the capacitor formed at p–n junction interface which defines the t_r_ and t_d_ values. Thus, the AZO/CuCrO_2_ photovoltaic configuration is much faster than the Au/AZO/Au planar structure due to the formation of very small capacitor at the p–n junction interface. We can also calculate the photosensitivity of the devices from these figures, which is another key factor in the evaluation of photodetector performance. Photosensitivity is defined as the ratio of photogenerated current (I_ph_) to the dark current (I_d_). In our fabricated devices, J_ph_ = 56 nA/cm^2^ and 346 µA/cm^2^ and J_d_ = 2.4 and 8.36 nA/cm^2^ at V = 1 V biasing voltage, giving photosensitivity ratios of ~ 24 and ~ 41,000, respectively for photoconductive and photovoltaic configurations. We see that the fabricated p–n junction enhances the photosensitivity in three orders of magnitudes. Of course, this increase in the photosensitivity is due to the lower dark current density of the p-n device rather than its increased photogenerated current. The reduction in the dark current from the photoconductor to photodetector configuration is performed as a result of depletion layer formation in the p–n junction, which forbids the majority carriers to be conducted through the device.

The most influential parameters of the photodetector devices are their responsivity (R) and detectivity (${D}^{*}$). In other optoelectronic devices such as solar cells, quantum efficiency, which is defined as the optical to electrical power ratio, is more familiar but it is more convenient to exhibit the efficiency of the photodetectors with their responsivity, which is defined as the ratio of output current to the input optical power. In photodetectors, this parameter is strongly dependent on the wavelength of the incoming light, and thus we have examined it for our devices in different wavelengths that are shown in Fig. 3f. In these spectra, which are acquired at zero bias condition, we observe a peak at UV wavelengths in both devices. This is a result of the optical absorption of the employed materials, which is high in the UV region and demonstrates the excellent selectivity of our fabricated UV photodetectors. The values of responsivity for the photoconductive and photovoltaic configurations at 385 nm wavelength are respectively 0.655 and 1.645 mA/W. We can clearly see that the proposed photovoltaic configuration has a much superior responsivity than its photoconductive counterpart, which is due to the improved charge separation in the p–n junction and reduced recombination of the charge carriers. Furthermore, our proposed device demonstrates outstanding performance in comparison with the previous similar thin-film UV photodetectors such as the ones proposed in the works of Tonooka et al. with Mg-doped CuCrO_2_ as the p-type and ZnO as the n-type materials^65^ or Bakar et al. with CuGaO_2_ and ZnO thin films^66^. We can see in Fig. 3f that the amount of responsivity decreases in both devices for longer wavelengths. Therefore, we can expect low optical noises in the photodetectors at ambient lighting.

Detectivity, which can be regarded as the photodetectors’ figure of merit is calculated from:

$${D}^{*}=\frac{R}{\sqrt{2q{J}_{dark}}}$$

in which the detectivity is in the Jones unit ($\frac{cm\sqrt{Hz}}{W}$) and $q$ refers to the elementary charge. We calculated the detectivity of our samples at 1 V biasing voltage to be 1.0 × 10^10^ and 3.5 × 10^12^ Jones that once again shows the superiority of our proposed photovoltaic UV photodetector to the typical photoconductor configuration. Comparing our work to some recent thin-film UV photodetectors, we can name the studies of Debnath et al. and Patel et al., who respectively attained detectivities of 6.3 × 10^11^ and 7.2 × 10^11^ Jones^67,68^. In comparison, our structure demonstrates a considerable improvement in the detectivity.

In summary, we fabricated two AZO-based UV photodetectors in photovoltaic and photoconductive configurations using the sputtering method. In order to create a suitable p–n junction in the photovoltaic configuration, we employed the (Mg and N)-doped CuCrO_2_ as the p-type layer. This structure demonstrated a far superior performance in comparison with the photoconductive structures. Regarding the rise and decay times, our proposed structure showed t_r_ = 0.21 s and t_d_ = 0.26 s that were much faster than t_r_ = 2.61 s and t_d_ = 2.85 s values of the single AZO layer. The photosensitivity, responsivity, and detectivity parameters of these devices were ~ 24, 0.655 mA/W and 10 × 10^9^ Jones for the AZO device and ~ 41,000, 1.645 mA/W, and 3.52 × 10^12^ Jones for the AZO/co-doped CuCrO_2_ bilayer device. These excellent improvements in the photodetector characteristics are attributed to the formation of the depletion region at the p–n junction that generates a built-in voltage which facilitates charge separation and collection, and also to the appropriate selection of the employed materials that are suitable in case of band structure and conductivity.

## Methods

**Figure 4:**
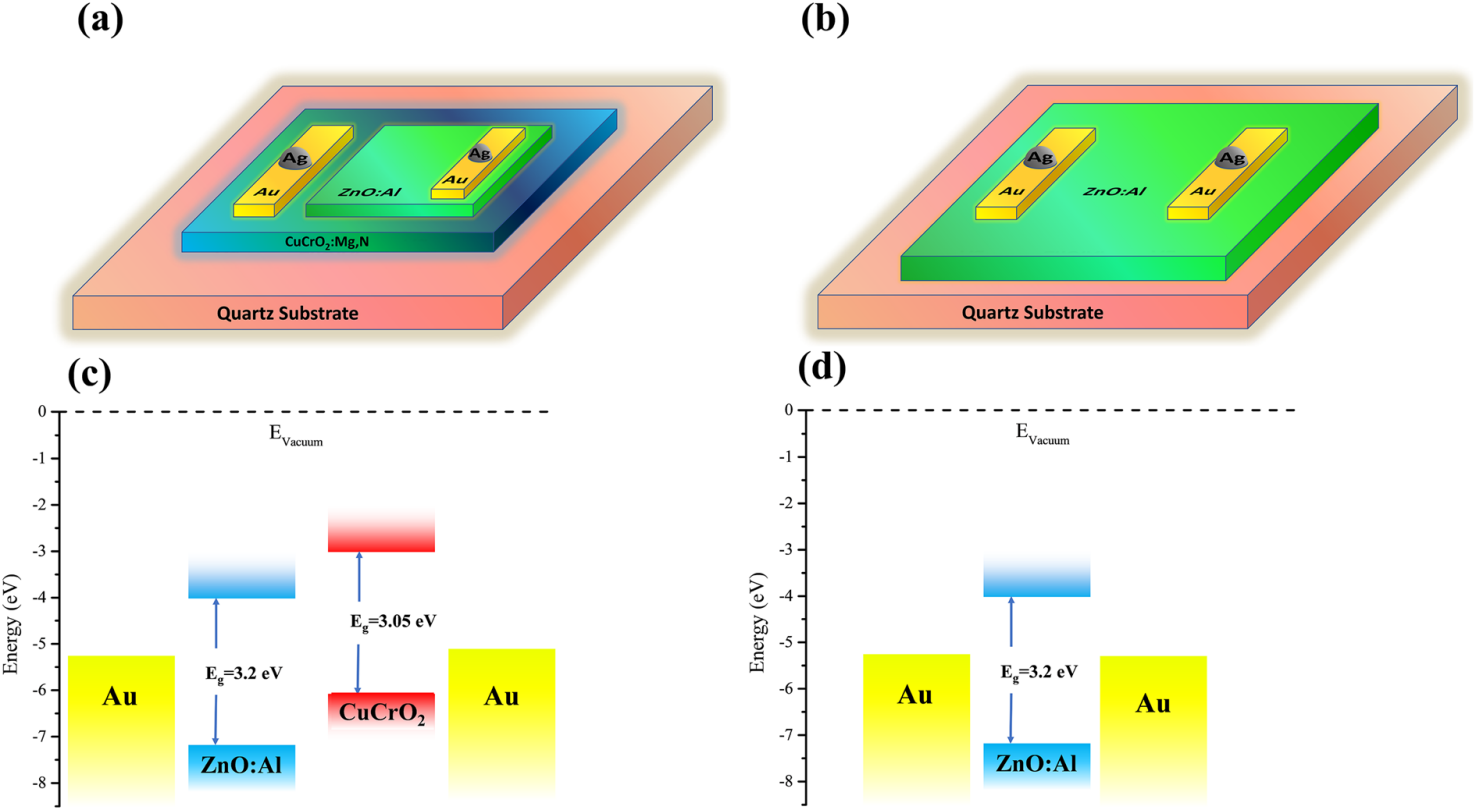
(**a**) Schematic presentation of the photovoltaic configuration with co-doped CuCrO_2_ as the p-type layer and AZO as the n-type UV absorber, all deposited on quartz substrate with Ag/Au ohmic contacts. (**b**) Schematic presentation of the photoconductive configuration that is composed of an AZO layer with Ag/Au contacts deposited on quartz substrate. [The schematics are created using Microsoft office PowerPoint 2019 software] (**c**) Band structure diagram of the photovoltaic configuration and (**d**) photoconductive configuration plotted with bandgaps of 3.05 eV^46^ and 3.2 eV^47^ and electron affinities^15^ of 3 and 4 eV, respectively for CuCrO_2_ and ZnO layers.

In order to fabricate the p–n junction required for the photovoltaic UV photodetector, we employed Mg and N co-doped CuCrO_2_ and Al-doped ZnO as p-type and n-type layers, respectively. We also fabricated a single AZO layer as a photoconductive UV photodetector. The schematics of our proposed devices are illustrated in Fig. 4 along with their estimated band structure, which shows a type II band alignment for the heterojunction. In these devices, we employed 2 cm × 2 cm flat quartz glasses as substrate. All the other layers were deposited using the sputtering method. A silver paste (EM-Tec AG42) was used to attach silver wires to the devices to complete the photodetector device fabrication.

For the preparation of the co-doped CuCrO_2_ layer, we first prepared an Mg-doped CuCrO_2_ ceramic target using the solid-state reaction method. For this purpose, stoichiometric ratios of CuO, Cr_2_O_3,_ and MgO powders (analytical grade, Merck Co.) were dispersed in HPLC ethanol (Merck Co.) and milled in a planetary ball milling system (NARYA MPM-2 × 250 H, Amin-Asia Co.) for 20 h (with 10 min intervals and 10 min rest times) with a speed of 400 rpm. The resulting mixture was dried at room temperature and then pressed into a 5 cm pellet using a hydraulic press with 40 MPa pressure. The pellet was sintered in an electric furnace at 1250 °C for 12 h. To deposit the co-doped thin films, the target was placed in a sputtering system along with the quartz substrates that were previously washed ultrasonically using acetone, ethanol, and deionized water at a distance of 5 cm from the target. The surroundings of the substrates were covered by aluminum square masks to prevent their lateral faces from being deposited. We evacuated the chamber to a base pressure of 3 × 10^–5^ mbar using Pfeiffer diaphragm and turbomolecular pumps. To initiate the plasma, we increased the pressure to 1.2 × 10^–1^ mbar by inserting a mixture of Ar and N grade 5 gasses into the chamber and applying an RF power of 150 W. Then, we reduced the pressure to 5 × 10^–3^ mbar in which the sputtered thin film has the highest quality^44^ and deposited a ~ 100 nm layer of co-doped CuCrO_2_ while heating the substrates with a temperature of ~ 400 °C for better crystallinity and incorporation of N into the delafossite lattice. We also performed a post-deposition annealing step on the (Mg, N)-doped CuCrO_2_ layers in a vacuum furnace with 2 × 10^–5^ mbar pressure at 900 °C for 2 h.

In order to prepare the AZO targets, we mixed ZnO (Merck Co.), and Al_2_O_3_ (Merck Co.) powders with a weight ratio of 98:2 and milled it with the same procedure of CuCrO_2_ target mentioned above. The resulting powder was calcined in the air at 1250 °C for 5 h. The obtained powder was ground again with the same ball milling process to achieve small and homogeneous particles. Then, the powder was pressed into a 5 cm diameter pellet at a pressure of 30 MPa, and finally, the developed pellet was re-sintered at 900 °C for 10 h under 2 × 10^–2^ mbar pressure. The target was placed in the sputtering system. This time we used two different substates, first an ultrasonically cleaned quartz flat glass and second the co-doped CuCrO_2_ thin film on quartz with an aluminum mask which covered the surroundings of the thin film. We deposited a 100 nm AZO layer using the RF sputtering technique with 150 W power and at 3.3 × 10^–3^ mbar pressure on the substrates that were rotating with 10 rpm speed for better uniformity of the thin films. No post-deposition annealing was performed for the AZO layers.

To achieve ohmic contact with the layers, we used another mask that only allowed deposition on the desired contact places. We deposited successive layers of Au and Ag using the DC sputtering method and attached Ag wires to them by means of the Ag paste. The contacts were heat-treated using a hot-air solder with 400 °C temperature to remove the Ag paste's organics and for better diffusion of metals at the metal/semiconductor interfaces.

The crystallographic analysis of our samples was carried out by a PW 1730 grazing X-ray diffractometer (XRD). We investigated the optical properties of the samples by an AvaSpec-3648 UV–visible (UV–vis) spectrometer. The morphologic data of the prepared thin films were examined by a TESCAN Mira 3 xmu field emission electron microscope (FESEM), and the elemental information was acquired by an Oxford 80-XMAX energy dispersive X-ray spectrometer (EDS). To analyze the materials’ structure, we also employed a Teksan, Takram P50C0R10 Raman spectrometer. The electrical parameters and transient behavior of our fabricated devices were investigated using a Tektronix high-resolution Keithley source meter.

## Supplementary Information

Supplementary Information.
