# Supplementary material for: An all-sputtered photovoltaic ultraviolet photodetector based on co-doped CuCrO2 and Al-doped ZnO heterojunction
Source: Sci Rep. 2021 Sep 21;11:18694. doi: 10.1038/s41598-021-98273-5 (PMC8455524; doi:10.1038/s41598-021-98273-5)
Supplement: Supplementary file 1 — Supplementary Information. [file 41598_2021_98273_MOESM1_ESM.docx]

**(Supplementary Information)**


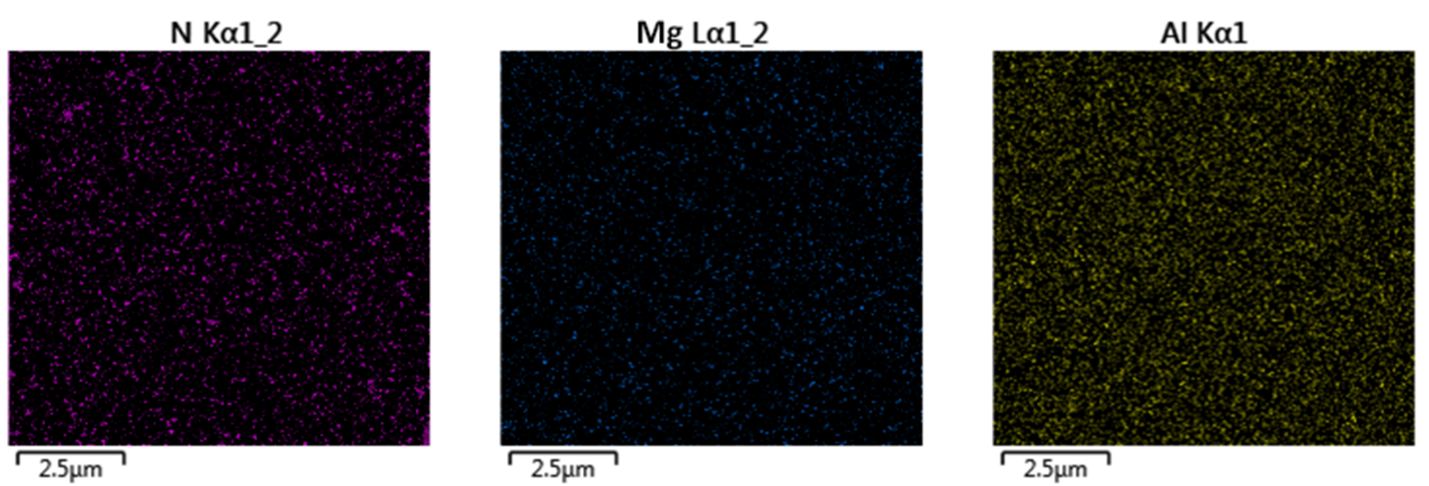


Fig.S1: EDS mapping analysis of the dopants in the prepared thin films.

Table S1: EDS quantification of the AZO and (Mg, N)-doped thin films.

| Element (At%) | Zn | O | Cu | Cr | O | Al | Mg | N |
| --- | --- | --- | --- | --- | --- | --- | --- | --- |
| Al-Doped ZnO | 54.7 | 43.2 | --- | --- | --- | 2.1 | --- | --- |
| (Mg,N)-Doped CuCrO_2_ | --- | --- | 24.2 | 21.8 | 44.2 | --- | 1.3 | 8.5 |


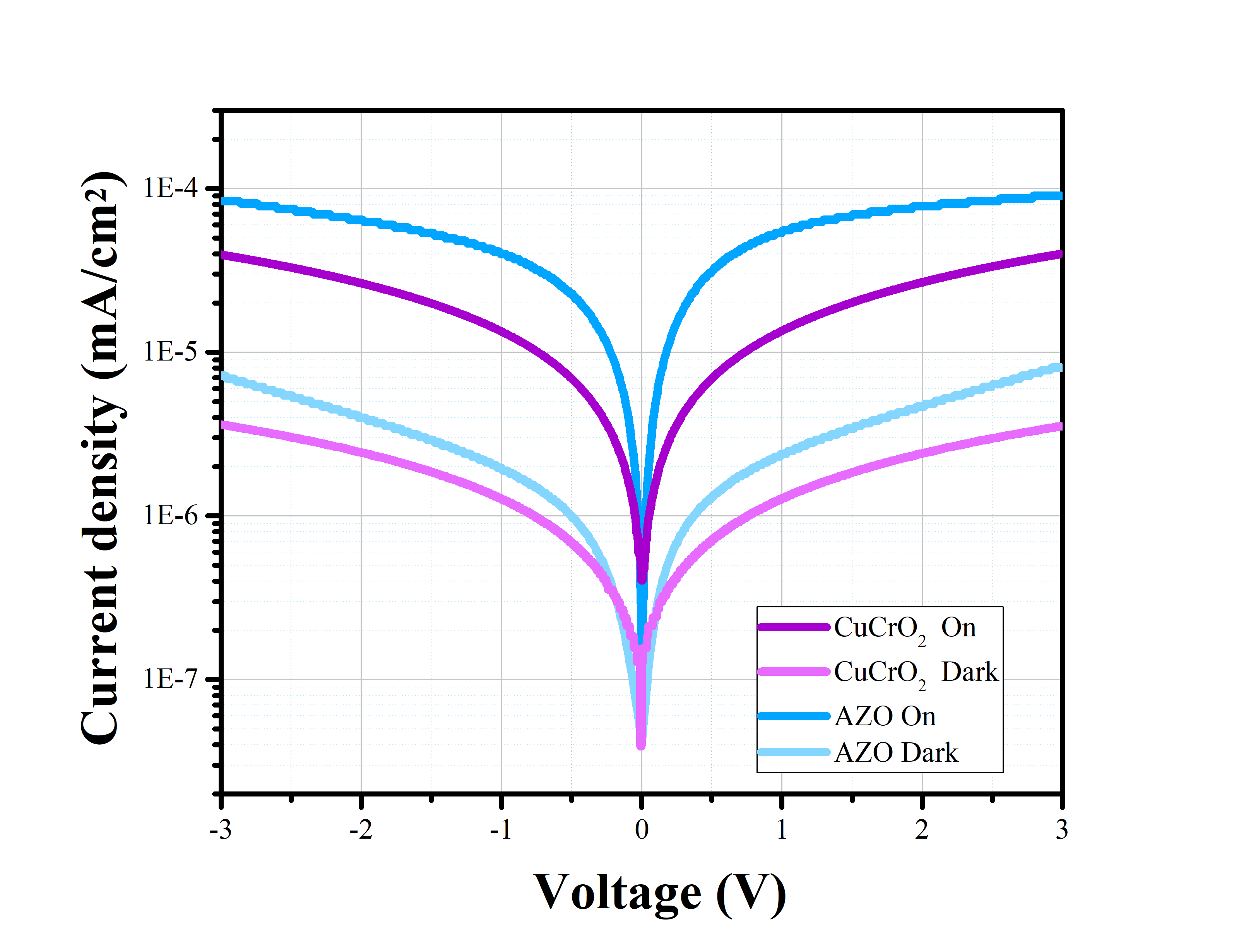


Fig.S2: Semilogarithmic plot of the J-V characteristics of the AZO and co-doped CuCrO_2_ photoconductive photodetectors in dark and illuminated conditions. The lower performance of the p-type CuCrO_2_ material in comparison with the AZO n-type layer is quite clear in this figure.


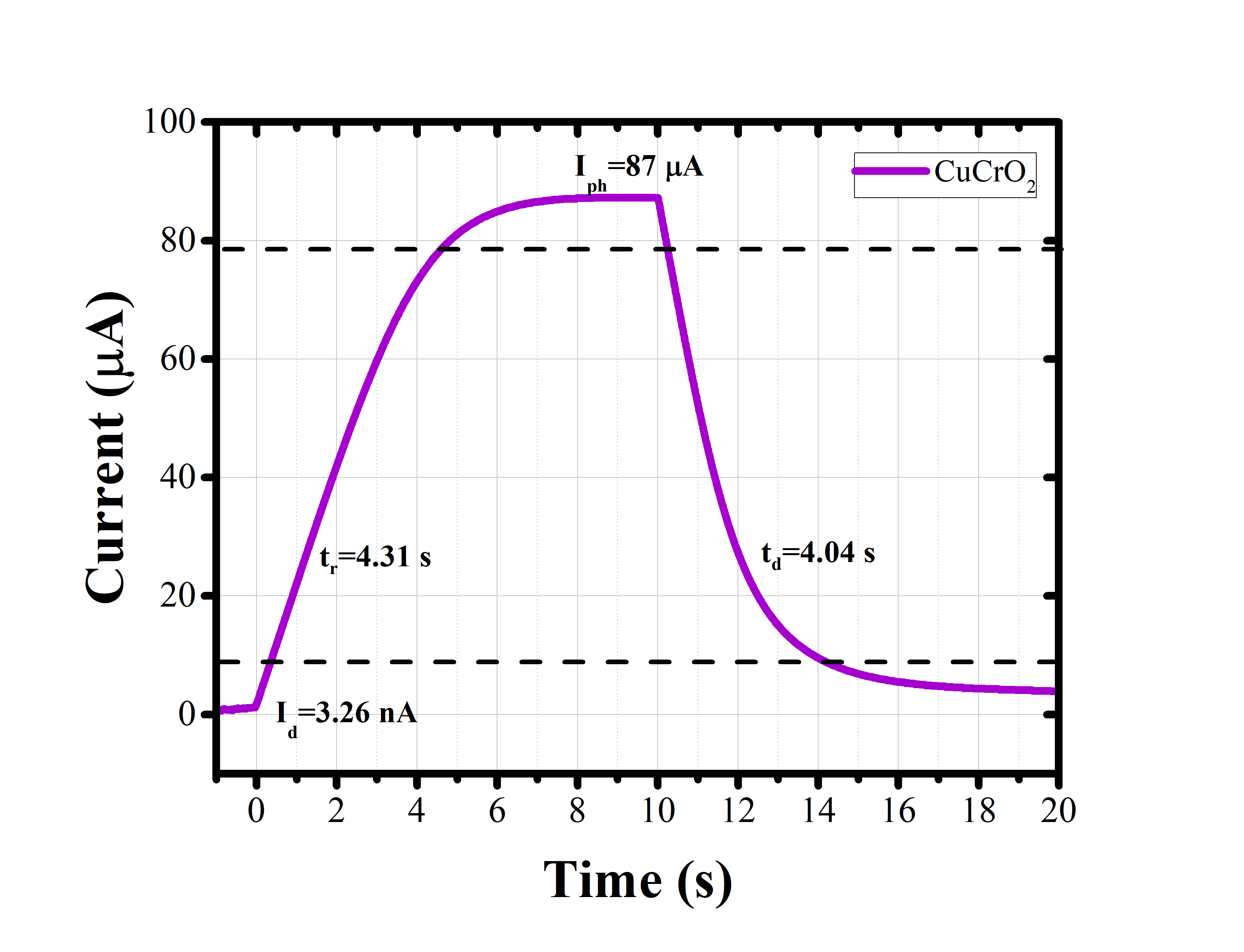


Fig.S3: Transient behavior of the co-doped CuCrO_2_ layer under UV illumination.
